# Editorial: Dietary and nutritional indices and chronic diseases

**DOI:** 10.3389/fnut.2023.1214617

**Published:** 2023-05-24

**Authors:** Sorayya Kheirouri, Mohammad Alizadeh, Masayo Nakamori-Rossignoli

**Affiliations:** ^1^Faculty of Nutrition and Food Sciences, Tabriz University of Medical Sciences, Tabriz, Iran; ^2^Department of Nutrition and Food Science, Kyoto Kacho University, Kyoto, Japan

**Keywords:** dietary indices, nutritional indices, dietary patterns, chronic diseases, nutrients

## Introduction

Regular or disease-specific diets are composed of different components, each of which has different properties such as high and low inflammatory, oxidative, acidogenic, glycemic and insulin load, etc. Therefore, the cumulative health effects of the food items may significantly differ on the body. Each of these food properties can be used as a specific index that can be linked to proper health outcomes. To measure each index, the attribute is calculated for all food components and then summed up to constitute the general characteristics of a diet in that aspect. In this regard, we established our Research Topic on March 28, 2022, and invited researchers interested in the benefits, concerns, or harms associated with diets of different characteristics with the greatest emphasis on prevention, control, and management of chronic diseases.

Frontiers in Nutrition published 20 articles that evaluated the association of dietary indices (*n* = 8), nutritional indices (*n* = 2), dietary patterns (*n* = 2), and specific nutrients or food groups (*n* = 8) with various health outcomes. Most of the studies were prospective cohorts or derived from cohort studies with large sample sizes.

## Dietary indices and health outcomes

Valle-Hita et al. in a prospective cohort study on 9,513 older adults with metabolic syndrome found that higher dietary acid load was associated with lower kidney function after 1 year of follow-up.

Nie et al., in a cohort study on 23,109 adult people, reported that individuals with the highest Healthy Eating Index 2015 (HEI-2015) score had a 12.2% reduced risk of gout and a 2.2% reduced risk of hyperuricemia than individuals with the lowest HEI-2015 score.

Moludi et al., in a study derived from the RaNCD cohort survey involving 9,824 individuals, reported that a high intake of a pro-inflammatory diet was related to a higher incidence of chronic kidney disease.

## Nutritional indices and health outcomes

Wang J. et al., in a study on 13,871 adults, found a positive association between visceral adiposity index with urolithiasis and the prevalence of kidney stones.

Han et al., in a retrospective cohort study on 4,411 patients with heart failure, showed that a high triglyceride glucose index was significantly correlated with the risk of in-hospital mortality in the patients, independent of type 2 diabetes mellitus and coronary artery disease.

## Dietary patterns and health outcomes

Llaha et al., in a prospective cohort study on 450,000 individuals from nine European countries, concluded that high adherence to the Mediterranean diet was not strongly associated with differentiated thyroid cancer risk after 14.1 years of follow-up. The authors found that low meat and moderate alcohol intake were associated with lower differentiated thyroid cancer risk.

Wang Y. B. et al., in a cross-sectional study on 1,792 community-dwelling adults, showed that a plant-sourced nutrient pattern was strongly and independently related to lower systemic inflammation, particularly in men and obese individuals.

## Specific nutrients or food groups and health outcomes

Tao et al., in a Mendelian randomization study on 5,575 participants, found a causal relationship between n-3 PUFAs, n-6 PUFAs, the ratio of n-3 PUFAs to total fatty acids, the ratio of n-6 PUFAs to n-3 PUFAs with estimated bone mineral density. The authors also showed an association between n-3 PUFAs with forearm and lumbar spine with bone mineral density and fracture.

Li Q.-H. et al., in a study on 655 gout patients, determined the high intake of sugar-sweetened beverages as the main dietary risk factor for gout in early-onset patients and found a direct association between sugar-sweetened beverages with serum urate level and obesity.

Peng et al., in a study of 7,725 participants, concluded that intake of a diet with a low percentage of energy from fat appears to be beneficial in the prevention of osteoarthritis risk.

Huang et al., in a study on 2,533 normotensive individuals, reported that serum vitamin C was adversely associated with both systolic and diastolic blood pressure.

All the investigations, except one, comprised in this Research Topic reinforced a link between healthy diet/nutrition status and improvement of health consequences. Overall, the findings of the articles published in this Research Topic may be beneficial in understanding that the dietary/nutritional indices could be effectively used as predictive biomarkers of health outcomes, particularly in patients with chronic diseases. Further, the evaluation of the indices would be beneficial in providing the necessary recommendations for the promotion of dietary status and in the consequent improvement of the health status of people, either at the clinical or community level.

Definitely, the number of 20 studies included in the present Research Topic is not able to fully cover all aspects of the Research Topic. The areas that were less addressed in the current Research Topic are:

- Interventional studies confirming the causal relationship between dietary/nutritional indices-health outcomes,
- Mechanistic pathways linking dietary/nutritional indices and chronic diseases,
- Studies linking dietary/nutritional indices with metabolic status, acid-base balance, inflammatory and oxidative stress conditions in the body, circulating biomarkers, and risk factors.

## Author contributions

SK prepared initial draft of the article. MA involved in the revision of the article. All authors contributed to the article and approved the submitted version.

